# How and When Daily Abusive Supervision Affects Daily Organizational Citizenship Behavior for the Environment

**DOI:** 10.3389/fpsyg.2022.791803

**Published:** 2022-05-27

**Authors:** Hongqing Wang, Jiuling Xiao

**Affiliations:** School of Business, Nanjing Audit University, Nanjing, China

**Keywords:** abusive supervision, organizational citizenship behavior for the environment, moral disengagement, psychological green climate, daily diary study

## Abstract

Organizational environmental sustainability depends primarily on employees’ organizational citizenship behavior for the environment (OCBE), and leadership plays an important role in improving and inhibiting employees’ OCBE. The purpose of the present study is to examine the mediation process by which abusive supervision affects employees’ OCBE through a daily diary study and to explore the boundary conditions of the relationship between daily moral disengagement and daily OCBE. We collected data from 112 Chinese employees for 10 consecutive days. The results show that daily abusive supervision has a significant negative effect on daily OCBE and that daily moral disengagement plays a significant mediating role in this relationship. In addition, the negative effect of daily moral disengagement on daily OCBE could be attenuated by a psychological green climate. Our findings support our hypotheses and offer useful theoretical and practical implications for promoting OCBE.

## Introduction

In the context of today’s growing local and global concerns about significant environmental issues, employee environmental actions are regarded as one of the main ways in which organizational environmental performance and environmental sustainability development can be improved; these sustainability behaviors are broadly referred to as employee pro-environmental or green behaviors and defined as “behaviors that employees engage in that are linked with and contribute to environmental sustainability” (Ones and Dilchert, 2012, p. 87; Wang et al., 2018; Sabbir and Taufique, 2021). Employee pro-environmental behavior can be divided into task-related (required) and proactive (voluntary) behaviors, and these behaviors differ in whether they are included in a formal role or a part of an organization’s requirements (Lülfs and Hahn, 2013; Norton et al., 2015; Wu et al., 2021). The success of an organization’s important environmental projects depends on voluntary pro-environmental behavior, which is not required by formal management systems (Boiral and Paillé, 2012). Boiral (2009) defines these voluntary behaviors as organizational citizenship behavior for the environment (OCBE), which is defined as “individual and discretionary social behaviors that are not explicitly recognized by the formal reward system and that contribute to a more effective environmental management by organizations” (p. 223); such behaviors include recycling, turning off electric appliances when not in use, preferring to use stairs instead of elevators, using public transportation, and drinking from reusable cups and bottles (Bissing-Olson et al., 2013; Dumont et al., 2017; Wang et al., 2017; Yuriev et al., 2018; Mi et al., 2019; Anser et al., 2021; Chen et al., 2021). OCBE makes significant contributions to organizational environmental performance and competitive advantage (Del Brio et al., 2007; Jackson et al., 2012; Boiral et al., 2015); thus, scholars have become increasingly interested in predicting employees’ level of OCBE (Bissing-Olson et al., 2013). Leadership has been highlighted as a key antecedent affecting employees’ OCBE (Robertson and Carleton, 2018; Khan et al., 2021; Wang et al., 2021).

Scholars have explored the effects of different positive leadership styles, such as servant leadership, transformational leadership, empowering leadership, responsible leadership, ethical leadership, and spiritual leadership, on employees’ OCBE (Afsar et al., 2016; Mi et al., 2019; Afsar et al., 2020; Raza, 2020; Cheng et al., 2021; Khan et al., 2021). However, the influence of leadership on employees’ behavior is not always positive (Choi et al., 2019); a growing body of evidence indicates that leaders may engage in destructive leadership, “which is a broad construct that captures styles of leadership comprised of behaviors embedded within leadership influence processes that harm followers and/or organizations” (Mackey et al., 2021, p. 705), such as abusive supervision, exploitative leadership, toxic leadership, and evil leadership (Aasland et al., 2010; Zhang and Liao, 2015; Milosevic et al., 2020). Studies have shown that there is a great difference in how individuals process positive and negative information (Schmid et al., 2018); specifically, negative information or behavior often has a stronger and more enduring impact than positive information (Baumeister et al., 2001). Although we know much about the impact of positive leadership on OCBE, less is known about the effect of destructive leadership on OCBE. As a typical form of destructive leadership, abusive supervision has attracted the greatest amount of attention in the organizational behavior research field (Zhang and Liu, 2018; Zappalà et al., 2022); therefore, we will focus on the effect of abusive supervision on OCBE.

As a form of immoral behavior, abusive supervision violates moral standards and seriously affects employees’ ethical behavior within an organization (Park et al., 2018). Moral disengagement theory provides an explanation mechanism for their relationship; employees’ negative experiences of contextual factors may activate individuals’ moral disengagement, which in turn guides good and bad behavior (Detert et al., 2008; Khan et al., 2019). Moral disengagement refers to an individual’s ability to deactivate moral self-regulation and self-censure, which allows individuals to engage in behavior that is inconsistent with moral standards without the associated self-sanction and guilt (Samnani et al., 2014). As a cognitive defense and justification mechanism (Zhao et al., 2021), moral disengagement is activated when individuals are in stressful job situations (Fida et al., 2015). Previous studies have found that moral disengagement is an important cognitive mechanism that is used to explain an individual’s behavioral decisions when he or she encounters negative treatment (Rice et al., 2020; Zhao et al., 2021). Therefore, moral disengagement may provide a compelling theoretical foundation for understanding the inhibiting mechanism of OCBE in abusive supervision situations.

Moral disengagement theory emphasizes the role of situational factors in the expression of moral thoughts and actions; to be more specific, people should not only regulate their behaviors to align with their internal moral standards but also ensure that their behaviors conform to the moral expectations of their situation (Bandura et al., 1996; Huang et al., 2019). This is in line with the person–situation interaction perspective that OCBE is likely to be affected by personal and situational factors (Inoue and Alfaro-Barrantes, 2015). As a much-drawn organizational contextual factor, psychological climate plays a critical role in driving individual behavior (Rubel et al., 2021; Biswas et al., 2022). A specific psychological climate has a close effect on specific behaviors, such as the relationship between an ethical climate and ethical behavior (Wang and Xiao, 2021), and the relationship between an innovation climate and innovative behavior (Newman et al., 2020). Thus, it is necessary to consider the psychological green climate as a contextual factor when studying the formative mechanism of OCBE, which refers to employees’ perceptions and interpretations of organizational policies, procedures, and practices regarding environmental sustainability (Dumont et al., 2017). As stated above, we examine whether a psychological green climate could attenuate the negative effect of daily moral disengagement on employees’ daily OCBE.

Previous studies that investigated predictors of OCBE have largely focused on stable differences between individuals; however, there is accumulating evidence not only that employees differ from each other in their average or typical levels of OCBE but also that individual employees’ level of engagement in OCBE can vary substantially over time, for instance, across workdays (Bissing-Olson et al., 2013). Therefore, studies that focus solely on between-person factors may neglect an important source of variability in behavior, as within-person factors can explain a significant amount of variance in such behavior (Ohly et al., 2010). Therefore, there have been increasing calls for researchers to place greater emphasis on within-person variations in behavior, which can illuminate the factors associated with the emergence of behavior as it occurs (Norton et al., 2015). Furthermore, recent studies have found that the levels of abusive supervision and moral disengagement vary from day to day (Huang et al., 2017; Vogel and Mitchell, 2017). By integrating the within-and between-person approaches, the present study investigates the dynamic mechanism of daily abusive supervision affecting daily OCBE *via* daily moral disengagement and the role of psychological green climate as a between-person level boundary condition in the relationship between daily moral disengagement and daily OCBE.

The present research contributes to the environmental literature in the following ways. First, previous studies have demonstrated that positive leadership, such as spiritual leadership, responsible leadership, inclusive leadership, authentic leadership, and supportive leadership, has a significant effect on OCBE (Anser et al., 2021; Wu et al., 2021). While the destructive leadership associated with OCBE has not been studied in-depth, understanding the nature of these obstacles might shed more light on the success of environmental behaviors (Yuriev et al., 2018). This study examines the negative effect of abusive supervision on OCBE and reveals the leadership obstacles that impede employees from engaging in OCBE, which could help organizations overcome these obstacles. Second, this study used the daily diary method to examine the mechanism of abusive supervision on OCBE. Compared to the static approach, this approach could reduce retrospective bias and social desirability (Vogel and Mitchell, 2017). More importantly, it could explain the short-term variation in daily OCBE.

## Conceptual Framework and Research Hypotheses

### Daily Abusive Supervision and Daily Organizational Citizenship Behavior for the Environment

Abusive supervision refers to any display of hostile verbal or non-verbal behavior (excluding physical contact), and these behaviors are likely to vary on a day-to-day basis (Barnes et al., 2015). Employees attach importance to how they are treated by their leader; when individuals are criticized and ridiculed by abusive supervisors, they may feel that they have been treated unjustly or improperly and are not being treated with dignity or respect (Dedahanov et al., 2021; He et al., 2021). In response to this affront, subordinates are prone to punish an abusive boss by purposefully withholding additional efforts that would benefit the organization (Lyu et al., 2016; Johnson et al., 2020). One such response could logically be the withholding of volitional behaviors, such as organizational citizenship behavior, which are not a requirement of the job and could run counter to the goal of retaliation by making the supervisor’s job easier (Harris et al., 2011; Zhang and Liao, 2015). In the environmental literature, scholars have also confirmed that as a form of organizational citizenship behavior (Zientara and Zamojska, 2018), OCBE is also affected by the treatment experienced from supervisors (Paillé et al., 2020). Extant studies have found that employees are more prone to behaving responsibly toward the environment on the job if they perceive that their supervisors are supportive (Paillé et al., 2019; Paillé and Meija-Morelos, 2019). In contrast, when subordinates perceive their supervisors’ attitudes and behaviors as being unfavorable or unsupportive, they tend to withhold OCBE (Chen et al., 2021). In brief, supervisors’ style of support is crucial in influencing subordinates’ proactive involvement in environmental management; thus, OCBE can be considered a form of repayment in exchange for support (Paillé et al., 2013), while a lack of managerial support is a major impediment to employees’ environmental behaviors at work (Paillé and Mejia-Morelos, 2014). In this vein, as a non-supportive leader behavior (Usman et al., 2021), abusive supervision is expected to undermine employees’ motivation to be involved in OCBE because, in addition to offering less support, abusive supervision includes supervisors engaging in hostile and injustice behaviors toward employees.

*Hypothesis* 1: Daily abusive supervision has a negative effect on daily OCBE.

### Mediating Role of Daily Moral Disengagement

According to moral disengagement theory, moral disengagement includes three broad and eight specific interrelated mechanisms, namely, cognitively reconstructing unethical behaviors (moral justification, euphemistic labeling, and advantageous comparison), obscuring or distorting consequences (displacement of responsibility, diffusion of responsibility, and distorting consequences), and devaluing the target (dehumanization and attribution of blame) (Bandura et al., 1996; Samnani et al., 2014). Employees use a moral lens to process supervisors’ behavior (Low et al., 2019); thus, leaders have a key influence on the extent to which their subordinates habitually enact morally disengaged cognitions in the workplace (Moore et al., 2019). Repeated observations or experiences of unethical behaviors likely increase individuals’ moral leniency and forgetting of moral norms, which can result in the observer’s gradual moral disengagement, even without taking notice of this change, the situation may eventually reach a level where moral disengagement becomes the observer’s thoughtless routine behavior (Arain et al., 2021). Previous research has found that perceived victimization resulting from negative and unethical supervisory behavior, such as abusive supervision, might lead subordinates to feel dissatisfied with their supervisors or organizations, which in turn decreases their levels of moral self-regulation and increases their levels of moral violations, thereby ultimately triggering a moral disengagement process (He et al., 2019; Rice et al., 2020).

Moral disengagement may be an effective protection strategy for coping with abusive supervision (Fida et al., 2015) because it allows individuals to retaliate against their leaders and organizations without feeling guilty and distressed by reframing the related actions such that they no longer seem immoral (Huang et al., 2017). As such, moral disengagement may inhibit abused employees’ prosocial behaviors at work (Newman et al., 2019), such as their OCBE. For example, abused people could resort to distorting the consequences of their actions to sanitize the harm they cause, thus reducing their feelings of distress. In the context of OCBE, people argue that their personal environmental behavior is negligible in terms of causing significant harm to the environment (Wu et al., 2020). As stated above, on a given day, employees who report having been subjected to more abusive supervisory behavior tend to also report having relatively high levels of moral disengagement, which in turn makes employees less likely to engage in OCBE.

*Hypothesis* 2: Daily moral disengagement mediates the negative relationship between daily abusive supervision and daily OCBE.

### Moderating Role of Psychological Green Climate

As mentioned above, moral disengagement is a major internal impediment to employees’ pro-environmental behaviors; in addition, according to moral disengagement theory, individual behavior is regulated not only by internalized moral factors but also by special situational factors (Inoue and Alfaro-Barrantes, 2015). That is, individuals should not only pay attention to weigh, calculate, and integrate morally related information to ensure that their behaviors do not cause guilt and distress but also perceive and interpret their work environment as operating in accordance with their perceptions of the organization’s policies, procedures, and practices (Zheng et al., 2019). A psychological green climate provides employees with cues that, in addition to pursuing economic benefits, organizations also pay attention to green-related decisions and behaviors (Dumont et al., 2017). A strong psychological green climate means that their organization values and advocates employees’ environmental behaviors, i.e., that it is valuable, appropriate, and desired to engage in OCBE in their organization (Norton et al., 2014). Thus, in this context, although employees are able to withhold OCBE without suffering from guilt and distress, they are also less inclined to make this choice because in such an organization, employees commonly share the value of environmental behaviors; thus, engaging in OCBE can not only make a good impression on the organization but also relieve stress caused by cognitive dissonance (Norton et al., 2017; Huang et al., 2019). In a low-level psychological green climate, organizations express less concern about employees’ pro-environmental behaviors, and the psychological green climate is no longer the major consideration factor of employees’ OCBE decisions. Thus, their behavior decisions are more dependent on individual moral disengagement. Therefore, the psychological green climate is higher, and the negative effect of moral disengagement on OCBE is weaker.

*Hypothesis* 3: A psychological green climate moderates the negative relationship between daily moral disengagement and daily OCBE.

## Materials and Methods

### Participants and Procedure

In this study, the daily diary method was used to collect data; that is, respondents were required to complete the same questionnaire once a day for 10 consecutive weekdays. Because respondents answer the same questionnaire every day in such an approach, it is easy for them to feel burnout and boredom, which reduces participants’ willingness to participate in the survey. Therefore, the daily diary survey approach usually employs abbreviated scales; that is, each variable in the questionnaire has no more than five items, and the item with the highest factor load and the best representation of the original construct is usually selected. Additionally, the selected item should have dynamic fluctuation (Ohly et al., 2010).

The utilized survey included two questionnaires, namely, questionnaires A and B. Questionnaire A mainly included between-individual variables, such as demographic characteristics and psychological green climate, while questionnaire B mainly included within-individual variables, such as abusive supervision, moral disengagement, and OCBE. Surveys were collected mainly through on-site distribution and collection, and participants were full-time employees in China. The respondents were drawn from four food service companies, three finance companies, and two production companies. According to the statistics, 209 people participated in the survey. Due to the long duration of the survey, some people dropped out during the process, and the effective number of questionnaires included in the study was 112 between-individual questionnaires. We used our sample size to conduct power analysis using G-power 3.1, and the results showed that by assuming a medium effect size = 0.25, and alpha = 0.05, the power could achieve 0.99.

### Measures

Participants indicated how they felt at that moment using a seven-point Likert scale ranging from 1 (strongly disagree) to 7 (strongly agree). Within-individual variables were measured using an abbreviated scale, while daily abusive supervision was measured with a short form of a three-item scale developed by Tepper (2000); a sample item is “Today, my supervisor put me down in front of others.” The Cronbach’s alpha for this scale, which was averaged across the study period, was 0.78. Three items from Moore et al.’ (2012) moral disengagement scale were used to measure daily moral disengagement. A sample item is “People can’t be blamed for doing things that are technically wrong when all their friends are doing it too.” The Cronbach’s alpha for this scale, which was averaged across the study period, was 0.89. Daily OCBE was measured using a shortened scale with three items derived from Boiral and Paillé’s (2012) scale; a sample question is “Today, I spontaneously gave my time to remind colleagues to pay attention to environmental protection at work.” The Cronbach’s alpha value for this scale, which was averaged across the study period was 0.80. The between-individual variable psychological green climate was measured with a four-item scale proposed by Norton et al. (2014): “Our company is worried about its environmental impact,” and the scale provided an acceptable reliability (α = 0.81).

### Analysis

Data collected in daily diary studies contained a hierarchical structure in which daily assessments were nested within participants. We used hierarchical linear modeling (HLM) to test our hypotheses. All between-person level variables were centered at the grand mean and all within-person level variables were centered at the group mean. SPSS and MPLUS statistical software were used for the preliminary analyses; we also used MPLUS to estimate the 95% confidence intervals of the indirect effect.

## Results

### Descriptive Statistics

Means, standard deviations, correlations, and intraclass correlation (1) for all variables are presented in Table 1. The results show that daily abusive supervision has a significant positive correlation with daily moral disengagement (*r* = 0.49, *p* < 0.01); furthermore, there is a significant negative correlation between daily abusive supervision and daily OCBE (*r* = −0.37, *p* < 0.01), and the correlation between daily moral disengagement and daily OCBE is significant (*r* = −0.38, *p* < 0.01). At the between-person level, psychological green climate is positively related to OCBE (*r* = 0.23, *p* < 0.01), and OCBE is aggregated by daily OCBE. The null model results show that approximately 30% of the variance in abusive supervision, moral disengagement, and OCBE could be explained by the within-individual variances and that approximately 70% of the variance could be explained by the between-individual variances. Thus, it is appropriate to use hierarchical linear modeling to analyze our data.

**TABLE 1:** Means, standard deviations, and correlations of the between-and within-level variables.

Variables	Mean	SD	1	2	3	4
**Day-level variables**						ICC(1)
1. Abusive supervision	2.63	1.13				0.68
2. Moral disengagement	3.00	1.57	0.49**			0.67
3. OCBE	5.21	1.09	−0.37**	−0.38**		0.64
**Person-level variables**						
4. Psychological green climate	4.58	1.02	−0.19**	−0.28**	0.23**	

**p < 0.01. ICC(1) represents intraclass correlation (1).

### Confirmatory Factor Analysis

Multilevel confirmatory factor analysis results show that the four-factor model fit the data satisfactorily [χ^2^ (103) = 340.13, *p* < 0.001, CFI = 0.92, TLI = 0.90, RMSEA = 0.05, SRMR_within_ = 0.06, and SRMR_between_ = 0.07]. Additionally, the four-factor model fit the data better than the three-factor model [χ^2^ (108) = 588.75, *p* < 0.001, CFI = 0.84, TLI = 0.80, RMSEA = 0.06, SRMR_within_ = 0.07, and SRMR_between_ = 0.10], the two-factor model [χ^2^ (111) = 1282.91, *p* < 0.001, CFI = 0.61, TLI = 0.52, RMSEA = 0.10, SRMR_within_ = 0.12, and SRMR_between_ = 0.12], and the one-factor model [χ^2^ (112) = 1387.56, *p* < 0.001, CFI = 0.57, TLI = 0.48, RMSEA = 0.10, SRMR_within_ = 0.12, and SRMR_between_ = 0.15]. The results indicate that the variables included in this study can be empirically discriminated from each other. In addition, we used two methods to check for possible common variance. First, the results of a one-factor test show that the fit indicators of the one-factor model do not reach the statistical requirements. Second, the unmeasured latent methods factor test was used to construct an unmeasured method variable, namely, common method variance, by loading all indicators of the within-person and between-person levels and then developed a five-factor model that included the four-factor model and common method variance (Podsakoff et al., 2012). The results show that the five-factor model [χ^2^ (85) = 301.05, *p* < 0.001, CFI = 0.93, TLI = 0.88, RMSEA = 0.05, SRMR_within_ = 0.06, and SRMR_between_ = 0.05] does not substantially improve the goodness of fit of the four-factor model. Thus, there is no serious common variance problem in the study.

### Hypothesis Testing

The results of multilevel modeling analyses are shown in Table 2. Model 1 shows that daily abusive supervision has a significant negative effect on daily OCBE (β = −0.19, *p* < 0.01); thus, Hypothesis 1 is supported. Model 2 shows that daily abusive supervision is positively related to daily moral disengagement (β = 0.36, *p* < 0.01); when we include daily moral disengagement in the model, while the result of Model 3 shows that daily moral disengagement is significantly associated with daily OCBE (β = −0.15, *p* < 0.01), the effect of daily abusive supervision on daily OCBE changes from significant (β = −0.19, *p* < 0.01) to non-significant (β = −0.10, *p* > 0.05). In addition, the indirect effect of daily abusive supervision on daily OCBE through daily moral disengagement is also significant [95% CI = (−0.10, −0.02)]; thus, Hypothesis 2 is supported. Model 4 shows that daily moral disengagement and a psychological green climate have significant interactive effects on daily OCBE (β = 0.10, *p* < 0.01). In detail, the results of a simple slope analysis show that the effect of daily moral disengagement on daily OCBE is significant (β = −0.23, *p* < 0.001) when the level of psychological green climate is low, and the effect of daily moral disengagement on daily OCBE is non-significant (β = 0.02, *p* > 0.05) when the level of psychological green climate is high. The difference between these effects is also significant (β = 0.25, *p* < 0.01); thus, Hypothesis 3 is supported.

**TABLE 2:** HLM results of analyses predicting daily OCBE.

Variables	OCBE	Moral disengagement	OCBE	OCBE
	M1	M2	M3	M4
Intercept	5.14***	3.02***	5.21***	5.22***
**Day-level variables**				
Abusive supervision	−0.19**	0.36**	−0.10	−0.08
Moral disengagement			−0.15**	−0.14**
**Person-level variables**				
Psychological green climate				0.13
Moral disengagement* psychological green climate				0.10**
**Variance**				
σ^2^	0.35	0.51	0.29	0.29
τ_00_	0.67***	1.76***	0.58***	0.59***
τ_11_	0.29***	0.85***	0.19***	0.18***
τ_22_			0.12***	0.10***

***p < 0.001; **p < 0.01; *p < 0.05.

## Discussion

The purpose of this study was to examine the within-person inhibition effect of abusive supervision, the mechanism through which it operates to influence employees’ OCBE, and which organizational condition can attenuate this inhibition effect. The results show that it is necessary to use a cross-level design to study the predictors of OCBE because the variance in OCBE could be explained by both within-person (36%) and between-person variance (64%). We found that at the within-person level, daily abusive supervision has a significant negative effect on daily OCBE; that is, leader behavior may be an obstacle to impeding employees’ OCBE. Moreover, we reveal the dynamic mediation mechanism of daily abusive supervision affecting daily OCBE through daily moral disengagement. As a moral model for organizations, leader behavior influences employees’ moral standards; thus, leaders’ unfair treatment behavior may deactivate employees’ moral self-regulatory processes (Lian et al., 2020; Zhao et al., 2021) and discourage them from engaging in OCBE without feeling distress or guilt. Although daily moral disengagement could enable employees to withhold OCBE to prevent discomfort and self-condemnation, as reasonable individuals, employees should make their behaviors consistent with not only their internal moral standards but also their perceptions of their organization’s policies, procedures, and practices. This view is supported by our study that a psychological green climate can significantly attenuate the negative effect of daily moral disengagement on daily OCBE; that is, compared to employees with a stronger psychological green climate, employees who perceive a weaker psychological green climate are more likely to withhold OCBE due to moral disengagement.

### Theoretical Implications

First, given the important role of leadership in influencing employees’ OCBE, previous researchers have highlighted the role of leadership in motivating employees’ engagement in OCBE (Khan et al., 2021) and intensively investigated how positive leaders’ behaviors increase these prosocial behaviors. However, the extant research overlooks the negative effects of leadership behaviors on OCBE, even though it is also important to understand what kind of leadership (e.g., abusive supervision) might hinder these behaviors (Vogel and Mitchell, 2017) because abusive supervision is very common in organizations worldwide. As an immoral behavior, a previous study found that abusive supervision is a critical inhibition factor for employees’ voluntary behavior (Liu and Wang, 2013; Choi et al., 2019; Zhang et al., 2019). To better understand the formation mechanism of OCBE, scholars have called for examining the effect of abusive supervision on OCBE (Wu et al., 2021). Unfortunately, little is known about the effect and mechanisms of abusive supervision on employees’ OCBE. This study examines the impeding effect of daily abusive supervision on daily OCBE from the negative leadership perspective and simultaneously reveals the underlying mechanisms of daily abusive supervision on daily OCBE from a moral perspective. The present research enriches the research perspective on OCBE, which addresses the limitation that the relationship between leadership obstacles (abusive supervision) and OCBE has not been studied in depth. The findings of this study also enrich the existing knowledge regarding the mechanism of leadership behavior on employees’ OCBE.

Second, researchers have typically operationalized OCBE as a between-person variable (varies from person to person) and focused on temporally stable predictors (Norton et al., 2017); however, studies that focus solely on between-person factors may neglect short-term variability in behavior (Ohly et al., 2010). Recent research has found that within-person factors (vary within a given employee across time and situations) can explain a significant amount of variance in OCBE; however, employees’ daily OCBE and its predictors are not well understood (Bissing-Olson et al., 2013; Carfora et al., 2017). The present research examines the within-person effect of abusive supervision on OCBE through the mediating role of moral disengagement and discerns the cross-level moderating effect of the between-person factor (psychological green climate) on the relationship between daily moral disengagement and daily OCBE. The results show that approximately one-third of the total variance in abusive supervision, moral disengagement, and OCBE can be explained by within-person variance, whereas two-thirds of the variance can be explained by between-person variance. This study illustrates that it is necessary to use a multilevel approach to simultaneously explore the within-person and between-person predictors of OCBE.

Third, this study examines under which conditions the negative effect of daily moral disengagement on daily OCBE can be attenuated. The results demonstrate that the higher the level of perceived psychological green climate is, the weaker the within-person negative effect of moral disengagement on OCBE becomes. Our finding is consistent with previous studies that show that employees’ conduct is determined by not only individual factors but also work context factors, such as a psychological green climate, because such a climate can create a normative context, which then impacts the way workers behave (Roeck and Farooq, 2018; Zientara and Zamojska, 2018). Our findings respond to recent calls to understand whether the strength of employees’ perceptions of different organizational climates accentuates or attenuates the dynamic influence of moral disengagement on employees’ prosocial behavior (Newman et al., 2019) and to use a multilevel approach that considers both within-person and between-person factors to examine the formation mechanism of OCBE (Bissing-Olson et al., 2013).

Fourth, our study demonstrates that abusive supervision has a significant impediment effect on employees’ OCBE. Our research was conducted using a sample drawn from the Chinese cultural context; thus, the current research lacks the benefit of a cross-cultural sample. However, abusive supervision is prevalent in organizations worldwide; although employees’ perceptions and reactions to abusive management may be influenced by cultural context, previous research has found that the consequences of abusive supervision are similar across cultures (Liu and Wang, 2013; Xu et al., 2015). A recent meta-analytic review found no significant moderation effect in the relationship between abusive supervision and organizational citizenship behavior (OCB) (Zhang et al., 2019). Thus, as a special OCB, researchers in other countries could explore the inhibitive factor of OCBE from the abusive supervision perspective.

### Managerial Implications

First, this study finds that the more abusive behavior an employee experiences on a given day, the weaker his or her tendency to implement OCBE on that day is. Therefore, to effectively improve OCBE, organizations must take steps to reduce leaders’ abusive behavior. Because there are both within-individual and between-individual variances in abusive behavior, organizations can take the following steps. First, in the recruitment and selection process, organizations can use individual trait assessments to predict leaders’ abusive tendencies, such as authoritarian and hostile attribution styles, and then reduce abusive behavior at the source. Second, organizations can provide diversified leadership training to improve leaders’ comprehensive quality and management skills, improve leaders’ humanistic management thinking, and make leaders realize that they are employees’ servicers and that they should respect and care for employees. Third, organizations can establish safe complaint and punishment mechanisms so that employees can report their abusive experiences in a timely manner and management can then reduce abusive supervision behavior by increasing the cost of abusive supervision. Fourth, given the pervasiveness and inevitability of abusive supervision, organizations can resort to stress training to improve employees’ ability to copy with abusive behavior and prevent employees’ negative response to abusive behavior based on a tit-for-tat strategy.

Second, the results show that moral disengagement is an important dynamic mediating process through which daily abusive supervision affects daily OCBE; therefore, organizations should highlight the role of employee moral disengagement in predicting employee behaviors. Because moral disengagement can be explained by both within-person and between-person variance, organizations can take the following steps. First, as a state variable, employees’ moral disengagement may change over time because of supervisors’ abuse behavior; thus, organizations could mitigate subordinates’ propensity to morally disengage by encouraging managers to treat their subordinates ethically (Arain et al., 2021). As a trait variable, employees’ moral disengagement is relatively stable. Organizations can recruit, select, and retain job candidates with strong tendencies for trait-based moral self-regulation by conducting scenario simulation, moral trait tests, and other methods during the recruitment and selection processes (He et al., 2019). In addition, organizations can reinforce employees’ moral sensitivities and self-controlling capabilities and reduce the negative effect of unethical behavior on employees’ moral disengagement by conducting moral training and lectures, moral psychological counseling and guidance, and other ethics-focused education and training initiatives (Qin et al., 2020).

Third, this study signifies that psychological green climates can significantly weaken the inhibitory effect of daily moral disengagement on daily OCBE; thus, organizations should pay particular attention to establishing a psychological green climate. Organizations could dampen the cognitive and behavioral manifestations of high moral disengagement by developing more ethical climates characterized by policies, practices, and procedures that emphasize OCBE (Arain et al., 2021). For instance, organizations could repeatedly highlight positive examples of employee green behavior in company newsletters (Norton et al., 2017) or provide reputation incentives to employees who engage in environmental behaviors, such as awarding stars for environmental behavior every year. Organizations can take such measures to make employees realize that although OCBE is not a job responsibility, organizations both value and reward this behavior.

### Limitations and Future Research

This research has limitations. First, the variables used in this study were all self-reported by employees, which may increase the risk of common method variance and social desirability bias. Furthermore, while self-report measures are more appropriate for daily diary studies because this methodology involves brief and practical measures that must be administered over several days, the approach is are costly in terms of time and other resources and increases both the burden placed on participants and dropout rates (Ohly et al., 2010). Previous studies have confirmed that self-report measures are justified when examining abusive supervision and organizational citizenship behavior for the environment because individuals are most knowledgeable about their own behaviors and experiences (Vogel and Mitchell, 2017; Kirrane et al., 2018; Wang and Xiao, 2021; Xiao et al., 2021). The one-factor test, the unmeasured latent methods factor test, and the significant interaction effects found that common method variance is unlikely to be a serious concern in our study. In terms of social desirability bias, voluntary participation and anonymity can counter socially desirable response tendencies, and the extant empirical research reveals that social desirability has a low or nil effect on the way in which people report their organizational citizenship behavior for the environment and prosocial behaviors on anonymous questionnaires (Podsakoff et al., 2003; Norton et al., 2017; Lanz et al., 2021; Xiao et al., 2021). Nevertheless, to minimize the threat of common variance and social desirability bias, future studies could replicate our model by collecting data from different sources, such as supervisor ratings, peer ratings, and objective data.

Second, our respondents are all Chinese employees, although the theoretical logics and arguments were not culturally specific (Zheng et al., 2019). A previous review found that national cultures have a non-significant effect on the relationship between abusive supervision and OCB (Zhang et al., 2019). While we must be cautious in determining whether our findings are applicable to other cultural contexts, in order to improve the generalizability of our research conclusions, future research should reexamine our research model in different cultural contexts. In addition, the dropout rate (near 50%) was quite high, because we used paper questionnaires to collect the data, we entered only the data of the fully completed questionnaires into the computer, after which we did not save the paper questionnaires. As a result, we know very little about the differences between the original participants and the dropouts. Even though this may not pose a significant threat to the use of the diary research approach (Ohly et al., 2010), it is would be both important and interesting to understand more information regarding those participants who dropped out, for example, whether they quit because they did not want to waste paper. Therefore, future studies could repeat this study using both electronic and paper questionnaires, and analyze the characteristics of the dropouts.

Third, the diary method requires respondents to complete the same questionnaire for several consecutive days. To reduce employee burnout, increase employee participation and improve the quality of the questionnaire results, we constrained the questionnaire length by using abbreviated scales. Even though we chose items that measured the full set of behaviors, which is in line with prior daily research, and an extremely high correlation between shortened measures and the corresponding full scales has been previously confirmed, this use of this approach may nonetheless be viewed as a limitation (Harris et al., 2011; Qin et al., 2020). Therefore, future studies could appropriately extend the time interval of the questionnaire survey, for example, once a week for several weeks. Such an approach may not only weaken the respondents’ memory of the questionnaire but also alleviate respondents’ level of burnout. In addition, the full versions of the scales can be adopted in future studies to prevent the reliability and validity problems caused by using reduced scales.

## Conclusion

The purpose of this study was to explore the dynamic mechanism and boundary conditions of negative leadership behavior affecting employee environmental behavior by daily diary research, that is, at the within-person level, this study aimed to explore whether and how daily abusive supervision affects daily OCBE, while at the between-person level, the current study aimed to examine which conditions could attenuate the negative effect of daily abusive supervision on daily OCBE. The results indicate that daily abusive supervision has a significant negative effect on daily OCBE; i.e., on a given day, the more abusive behavior an employee experiences, the less likely he or she is to engage in OCBE. While previous studies have mainly emphasized the promoting effect of positive leadership behavior on OCBE, little is known about the relationship between abusive supervision and OCBE. Thus, our findings extend existing the knowledge on the relationship between leadership and OCBE. The current study reveals the dynamic processes through which daily abusive supervision influences daily OCBE and that leaders’ abusive behavior can trigger abused employees’ moral disengagement, which in turn can inhibit employees’ OCBE. In addition to individual factors, employee behavior is guided by the organizational climate. Our study confirms that psychological green climates can significantly attenuate the impeding effect of daily moral disengagement on daily OCBE. The higher the psychological green climate is, the weaker the inhibitory effect of daily moral disengagement on daily OCBE is. The results of this study offer implications for organizations that leadership behavior can also negatively impact employees’ OCBE, while a strong psychological green climate can help organizations motivate employees’ OCBE.

## Data Availability Statement

The raw data supporting the conclusions of this article will be made available by the authors, without undue reservation.

## Ethics Statement

The studies involving human participants were reviewed and approved by the Ethics Committee of Business School at Nanjing Audit University. Written informed consent for participation was not required for this study in accordance with the national legislation and the institutional requirements. Written informed consent was obtained from the individual(s) for the publication of any potentially identifiable images or data included in this article.

## Author Contributions

HW wrote the original draft of the manuscript and analyzed the data. JX contributed to data collection. Both authors contributed to the design and conceptualization of the manuscript and reviewed and edited the manuscript.

## Conflict of Interest

The authors declare that the research was conducted in the absence of any commercial or financial relationships that could be construed as a potential conflict of interest.

## Publisher’s Note

All claims expressed in this article are solely those of the authors and do not necessarily represent those of their affiliated organizations, or those of the publisher, the editors and the reviewers. Any product that may be evaluated in this article, or claim that may be made by its manufacturer, is not guaranteed or endorsed by the publisher.
